# Enhanced electrochemical properties of fluoride-coated LiCoO_2 _thin films

**DOI:** 10.1186/1556-276X-7-16

**Published:** 2012-01-05

**Authors:** Hye Jin Lee, Seuk Buom Kim, Yong Joon Park

**Affiliations:** 1 Department of Advanced Materials Engineering, Kyonggi University, Gyeonggi-do, 443-760, Republic of Korea

## Abstract

The electrochemical properties of fluoride-coated lithium cobalt oxide [LiCoO_2_] thin films were characterized. Aluminum fluoride [AlF_3_] and lanthanum fluoride [LaF_3_] coating layers were fabricated on a pristine LiCoO_2 _thin film by using a spin-coating process. The AlF_3_- and LaF_3_-coated films exhibited a higher rate capability, cyclic performance, and stability at high temperature than the pristine film. This indicates that the AlF_3 _and LaF_3 _layers effectively protected the surface of the pristine LiCoO_2 _film from the reactive electrolyte.

## Introduction

Lithium-ion batteries are used as power sources for a wide range of applications such as cellular phones, personal digital assistants [PDAs], laptop computers, and electric vehicles. The cathode is one of the critical components of a lithium-ion battery, and it determines the capacity, cyclic performance, and thermal stability of the battery. In order to improve the electrochemical properties of the cathode material, researchers have attempted to modify the cathode surface by using stable materials [1-5]. The coated cathode exhibits an enhanced rate capability, thermal stability, and cyclic performance. However, the coating effect is highly dependent on the material and shape of the coating layer [4,5]. Therefore, the identification of a suitable coating layer is a key factor in obtaining a highly improved cathode material by using the coating process. In this work, a fluoride-coated lithium cobalt oxide [LiCoO_2_] thin film was characterized. The surface of a LiCoO_2 _thin film cathode is much wider and smoother than that of a bulk-type electrode, which may enable careful observation of the interface reaction of a coating layer. Fluorides such as aluminum fluoride [AlF_3_] and lanthanum fluoride [LaF_3_] are promising coating materials for surface modification of the cathode [6-8]. Myung et al. proposed that a stable coating layer such as metal oxide transformed into a metal-fluoride layer during cycling, thereby leading to a greater resistance to HF attack [3]. This implies that the fluoride layer can be effectively used to protect the cathode surface from unwanted reactions with the electrolyte. Fluorine [F] has also been investigated for use as a doping material for enhanced structural and thermal stability [9-11]. In this study, we focused on the discharge capacity, rate capability, and cyclic performance of the pristine fluoride-coated LiCoO_2 _thin films to characterize the coating effect.

## Experimental details

The pristine LiCoO_2 _thin film was supplied by GS NanoTech Co., Ltd (Gangdong-gu, Seoul, South Korea). In order to prepare the AlF_3 _coating solution, aluminum nitrate nonahydrate (Al(NO_3_)_3_9H_2_O; Sigma-Aldrich, St. Louis, MO, USA) and ammonium fluoride [NH_4_F] (Sigma-Aldrich, St. Louis, MO, USA) were dissolved in 10 ml of a mixed solvent consisting of distilled water, 1-butanol, and acetic acid. The LaF_3 _coating solution was also prepared by dissolving lanthanum nitrate hexahydrate [La(NO_3_)_3_6H_2_0] and NH_4_F in a mixture of distilled water, 1-butanol, and acetic acid. The resultant solution was applied as a coating to the LiCoO_2 _thin film substrate by using a spin-coater (K-359 S-1, Kyowa Riken Co., Ltd., Tokyo, Japan). The coated LiCoO_2 _thin films were then heat-treated in a rapid thermal annealing [RTA] system at 400°C for 30 min. The microstructures of the films were observed by field emission - scanning electron microscopy [FE-SEM] (JEOL JSM-6500F, JEOL Ltd., Akishima, Tokyo, Japan). The electrochemical characterization of the coated LiCoO_2 _films was performed in non-aqueous half-cells. The cells were subjected to galvanostatic cycling using a galvanostatic system (WonATech, Seocho-gu, Seoul, South Korea).

## Results and discussion

**Figure 1:**
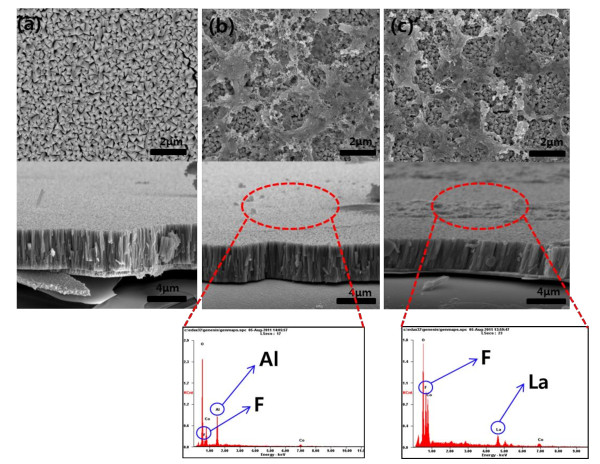
**SEM images of the pristine and coated LiCoO_2 _thin film electrodes**. (**a**) Pristine film, (**b**) AlF_3_-coated film, and (**c**) LaF_3_-coated film. The bottom of the figure shows EDS peaks of the surface of the coated samples.

Figure 1 shows the surface and cross-sectional images of the pristine and fluoride-coated LiCoO_2 _thin films. The surface of the pristine LiCoO_2 _film is composed of small polyhedral grains. As shown in Figure 1a, the crystal faces on the surface of the pristine film are very clear without any particles. In contrast, the coated film has a coarse, inhomogeneous surface morphology. As shown in Figures 1b and 1c, the coating layer consists of small nanoparticles. It appears that the AlF_3 _and LaF_3 _layers do not perfectly cover the surface of the LiCoO_2 _thin film. However, despite having a nonuniform coating layer, coated cathode powder generally presents a good coating effect [5-8]. The cross-sectional images of the samples are very similar. The thickness of the film cannot be measured from an SEM image, implying that the coating layer is very thin. However, the presence of Al, La, and F elements was confirmed by performing energy-dispersive spectroscopy [EDS] analysis of the coated film surface, as shown at the bottom of Figure 1.

**Figure 2:**
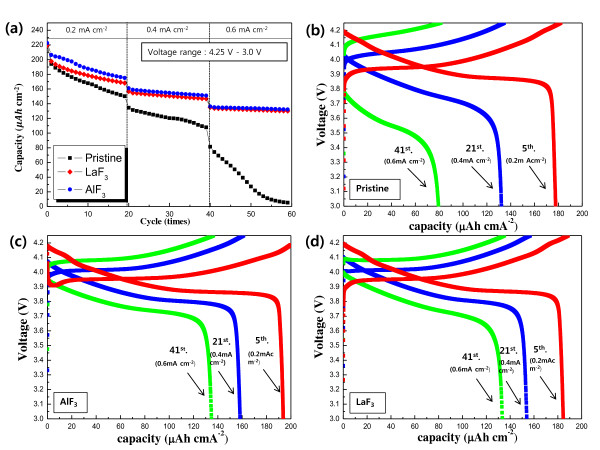
**Discharge capacities, cyclic performances, and discharge profiles of the thin films**. (**a**) Discharge capacities and cyclic performances of pristine and fluoride-coated LiCoO_2 _thin films, (**b**) discharge profile of the pristine film, (**c**) discharge profile of the AlF_3_-coated film, and (**d**) discharge profile of the LaF_3_-coated film. (The voltage was in the range of 4.25 to 3.0 V, and the current densities were 0.2, 0.4, and 0.6 mA· cm^-2^.).

The electrochemical properties of the pristine and coated LiCoO_2 _thin films were studied at various current densities (0.2, 0.4 and 0.6 mA· cm^-2^) in the voltage range of 4.25 to 3.0 V. In Figure 2a, the pristine and coated samples showed similar discharge capacities in the initial cycles. However, the discharge capacity of the pristine film dropped rapidly during cycling at higher current densities. The current densities in this study corresponded to cycling rates of 1, 2, and 3 C. The 4-μm-thick film electrodes do not contain conducting agents such as carbon, which render them vulnerable at high-rate cycling. In contrast, the rate capability and cyclic performance of the AlF_3_- and LaF_3_-coated films were superior to those of the pristine film. Figures 2b to 2d illustrate the voltage profiles of the pristine and coated samples at the current densities of 0.2, 0.4, and 0.6 mA· cm^-2 ^(the 5th, 21st, and 41st cycles are in Figure 2a). The capacity of the pristine film decreased sharply at high current densities; in contrast, the coated sample showed a greatly enhanced capacity retention. This indicates that the AlF_3 _and LaF_3 _coating layers improved the rate capability and cyclic performance of the LiCoO_2 _film. Generally, the cathode surface easily reacts with acidic electrolyte. This implies that dissolution of the transition metals in the cathode and formation of an unwanted layer could have occurred at the interface between the cathode and the electrolyte, which in turn could disturb the movement of lithium ions and electrons during cycling. The AlF_3 _and LaF_3 _coating layers are likely to effectively protect the surface of the LiCoO_2 _cathode film from the acidic electrolyte attack, thus preventing the deterioration of the cathode interface. This is possibly the reason for the enhancement of the rate capability and cyclic performance of the AlF_3_- and LaF_3_-coated films.

**Figure 3:**
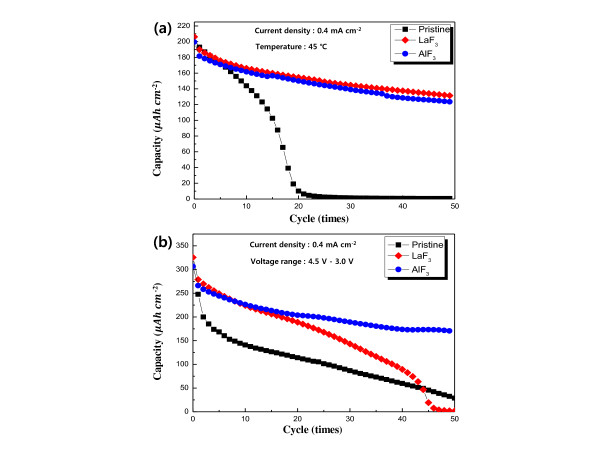
**Cyclic performances of the pristine and fluoride-coated LiCoO_2 _film electrodes**. (**a**) Cyclic performances measured at 45°C in the voltage range of 4.25 to 3.0 V at a current density of approximately 0.4 mA· cm^-2 ^and (**b**) measured at 30°C in the voltage range of 4.5 to 3.0 V at a current density of approximately 0.4 mA· cm^-2^.

To investigate the effects of AlF_3 _and LaF_3 _coatings under severe conditions, the pristine and coated samples were cycled at 45°C in the voltage range of 4.25 to 3.0 V (at a current density of 0.4 mA· cm^-2^). The high temperature activates the chemical reactions between the electrolyte and the electrode surfaces, thereby causing deterioration of the electrode. As expected, the discharge capacity of the pristine film showed a rapid fading effect. The discharge capacities of the AlF_3_- and LaF_3_-coated films also deteriorated during cycling. However, they showed greatly enhanced cyclic performances, as observed in Figure 3. This improvement may be attributed to the stable fluoride coating layer. The fluoride coating layer has been reported to offer high resistance to acidic electrolyte during cycling [3]. The stable AlF_3 _and LaF_3 _coating layers successfully prevented the unwanted reaction between the electrolyte and the interface layer in the cathode, leading to an enhanced cyclic performance under severe cycling conditions. Therefore, it is evident that both AlF_3 _and LaF_3 _are very effective coating materials that protect the cathode from deterioration during cycling at high temperatures.

The cyclic performances of the pristine and the AlF_3_- and LaF_3_-coated films were also investigated in the voltage range of 4.5 to 3.0 V (at a current density of 0.4 mA· cm^-2^). These are considered to be severe measurement conditions because LiCoO_2 _undergoes structural instability in the high voltage range (i.e., above 4.25 V) [12]. As shown in Figure 3b, all the samples showed a sharp drop in discharge capacities during several cycles. However, the AlF_3_- and LaF_3_-coated films showed a relatively moderate capacity fading. It is important to note that the AlF_3 _coating is more effective than the LaF_3 _coating in suppressing capacity fading in the high cutoff voltage range. This result indicates that AlF_3 _is a more effective coating material than LaF_3 _for increasing the structural stability of LiCoO_2 _in the high voltage range.

## Conclusions

Stable AlF_3 _and LaF_3 _coating layers were fabricated on a pristine LiCoO_2 _thin film electrode. The rate capability of the film electrode was evidently improved by the AlF_3 _and LaF_3 _coating layers. In particular, the coated film showed a greatly enhanced cyclic performance under severe cycling conditions. This indicates that the AlF_3 _and LaF_3 _coating layers were successful in preventing the surface of the LiCoO_2 _film from reacting with acidic electrolyte.

## Abbreviations

EDS: energy dispersive spectroscopy; FE-SEM: field emission - scanning electron microscopy; PDAs: personal digital assistants; RTA: rapid thermal annealing; SEM: scanning electron microscopy.

## Competing interests

The authors declare that they have no competing interests.

## Authors' contributions

HJ did the synthetic and characteristic works in this journal. YJ gave the advice and guided the experiment. SB helped in the SEM test. All authors read and approved the final manuscript.
